# Supplementary material for: Provider-initiated HIV testing and TB screening in the era of universal coverage: Are the right people being reached? A cohort study in Blantyre, Malawi
Source: PLoS One. 2020 Aug 13;15(8):e0236407. doi: 10.1371/journal.pone.0236407 (PMC7425889; doi:10.1371/journal.pone.0236407)
Supplement: Attachment — Submitted filename: Response to Reviewers.docx [file pone.0236407.s002.docx]

Dr Luke Mair MBChC, DTM&H
Department of Clinical Sciences,
Liverpool School of Tropical Medicine,
Pembroke Place,
Liverpool,
L3 5QA

Email: [Luke.mair1@nhs.net](mailto:Luke.mair1@nhs.net)
Mob: +447969239797

Dear Editors and Reviewers,

**RE: Provider-initiated HIV testing and TB screening in the era of universal coverage: are the right people being reached? A cohort study in Blantyre, Malawi**

I write on behalf of my co-authors to thank the reviewers for their careful review, and appreciate the opportunity to respond with a revised submission. Please see a point-by-point response below.

As recommended by the Reviewers, we have updated the manuscript to provide a more definite conclusion that the study shows poor implementation of national HIV testing guidelines and to emphasise that evaluation of additional strategies for increasing HIV testing rates are needed. The discussion has been expanded to further discuss potential strategies for improvement. This includes implementation of routine opt out HIV testing (ROOT), as well as the use of semi-supervised oral fluid-based HIV tests within facilities.

We hope that with the changes made as suggested by the reviewers this manuscript can be published and provide motivation for a change in HIV testing policy.

Yours sincerely,

Dr Luke Mair

**RESPONSE TO REVIEWER 1’S COMMENTS**

**Overall:**

Comment 1:

This manuscript describes a prospective cohort study undertaken in an adult primary care clinic in Blantyre Malawi to evaluate whether clinic attendees were offered PITC and to compare same day HIV testing rates between participants reporting TB symptoms vs those reporting no TB symptoms. From other data, rates of undiagnosed HIV are high among clinic clients reporting TB symptoms, therefore should be prioritized for HIV testing. The investigators relied on self-report of HIV-testing upon exit interview, and only 44% of clinic attendees underwent exit interview.

The main finding of the study was that among participants undergoing exit interview, only 27.6% reported being offered testing, with 21.5% completing PITC and receiving HIV test results. Testing offered was relatively similar among those with and without TB symptoms. The multivariable regression model indicated men, younger adults, and participants who had previously undergone HIV-testing were more likely to complete HIV testing than women, older adults, and those who had not previously been tested for HIV.

Thank you

Comment 2:

Although an important topic- universal HIV testing as well as importance of HIV testing among those with TB symptoms, the paper could be strengthened by a number of additions/clarification:

The authors do not included data regarding proportion of participants who report TB symptoms (and those without TB symptoms) who were diagnosed with HIV. This is an important data point and could be used to support their hypothesis that clinic goers with TB symptoms are a high yield population to test.

We thank the reviewer for these comments. The result of the HIV test was not asked of patients in this study. This paper focused on optimal testing strategies for HIV in patients with symptoms of tuberculosis. We relied on previous research undertaken in this field to show the known high prevalence of tuberculosis in patients with HIV symptoms. Therefore, allowing the reader to be aware that missed opportunities for testing were of high importance.

Comment 3:

Only 44% of clinic goers underwent exit interview. I wonder if it is possible to also reconstruct the cascade of testing and testing results utilizing the registers from the clinic over the same time period. Although these registers may not include the results of the TB symptom screen, they would provide an objective snapshot of number of clinic goers offered testing, undergoing testing, and testing results and would be very informative.

We thank the reviewer for these comments and agree that these additional sources of data would have been useful. Unfortunately however, as is common in extremely busy primary health care centres in Africa, routine documentation of clinic attendance in facility paper registers is poorly done. This was why, as part of the parent trial, we had to establish a biometric-based system for systematically recording clinic attendances and screening for TB. Given the unreliability of clinic registers, we wouldn’t be able to link to study data, and we don’t think that retrospective data extraction from these registers would provide reliable, useful information.

Comment 4:

Additionally, it appears clinic attendees with TB symptoms were less likely to undergo exit interview. The authors should clarify how this may have biased their results. Perhaps participants who had TB symptoms did under HIV testing, but they weren’t captured for some reason in the exit interview?

A limitation that is highlighted by the reviewer is that patients with TB symptoms were less likely to undergo exit interview than those without. We have reviewed these data carefully, and note that, in our analysis patients with any TB symptom other than chronic cough were **more** likely to undergo exit interview than those without. We have corrected this error, and have not discussed in the limitations section of the manuscript.

Comment 5:

Is the reason for clinic appt recorded? If so, would also include in the models of testing. Perhaps certain presenting complaints (or even primary diagnosis of the encounter) are related to testing offer, uptake, and diagnosis.

All participants attending the clinic did so because they had an acute illness (they were seen in the general outpatients department, where acute outpatient care is delivered). Given the high numbers of patients attending the clinic each day, we regret that it would not have been possible to systematically record or reliably classify reasons for clinic attendance.

**Introduction/Methods**

Comment 6:

Suggest clearly stating what the Malawian guidelines are for testing adults in a primary care clinic are.

Thank you for this suggestion. Malawian guidelines state that all patients attending outpatient departments should be offered PITC. In response an additional paragraph has been added to the introduction to highlight current Malawian National Guidelines.

*“Current Malawian National Guidelines focus on facility testing and community testing as priority opportunities for HIV testing and diagnosis. PITC is recommended for anyone attending healthcare facilities including outpatient departments as one of the most effective strategies for identifying new HIV diagnoses. Other strategies recommended include index testing and self-testing.”*

Comment 7:

Authors mention these visits are for acute care, but my assumption it is for all-comers. Please clarify

The clinic is structured so that people attending the clinic requiring acute outpatient care are triaged to the clinic outpatient waiting area. Other services available include outpatient HIV care clinic, TB clinic, antenatal care, and paediatrics; however clinic attenders requiring these services are directed to separate buildings within the clinic grounds. So, only adult patients attending the clinic with an acute illness were eligible for inclusion in the study. We noted this in the methods:

*“BHC has an adult outpatient department where community members can self-present and are registered and receive care free-of-charge for illnesses. Within BHC, there are additionally HIV, TB, antenatal, and paediatrics departments from where we did not recruit study participants.”*

**Methods**

Comment 8:

If possible, briefly describe what the pragmatic RCT will focus on.

An additional line has been added to methods section to provide an overview of the aims of the mentioned RCT. This addition will provide the reader with further knowledge and hopefully encourage readers to review the study protocol of this trial. In addition, we have referenced the published trial protocol.

*“This study was nested within the pilot phase of a pragmatic randomised trial being conducted at the clinic that aims to evaluate strategies to optimise diagnosis and treatment initiation for HIV and tuberculosis.”*

Comment 9:

Describe what the Malawian PITC guidelines are.

As above (Comment 6) this has now been included in the introduction section of the manuscript.

Comment 10:

Is TB care provided at the clinic, or are patients referred somewhere else. What are the next steps for patients with TB symptoms at the clinic? Perhaps if they are referred somewhere else this could account for the fact that they were difficult to reach for exit interviews

Thank you for this important comment. We have expanded the methodology section to include further description of the layout of Bangwe outpatient department and to describe the HIV and TB services available at Bangwe.

*“ART and antituberculosis therapy were provided onsite at the HIV care clinic and TB clinic respectively. Sputum smear microscopy and GeneXpert MTB/Rif testing were available onsite in the clinic laboratory. Patients requiring chest X-ray for investigation of possible tuberculosis required health worker referral to the city’s central hospital.”*

Comment 11:

Be consistent RE: capitalization for Research Assistant.

Thank you. This point has been addressed.

Comment 12:

Clarify if PITC is to be offered for those with a negative test >90 days, or unknown HIV status (these details aren’t mention- I’m just assuming this is the guideline).

WHO guidelines suggest that HIV testing be offered annually for those with a negative test in generalized HIV epidemics. The date of last test was not asked in this study. This has been included as a limitation of the study.

*“Thirdly patients were not asked the time since last HIV test in exit interview. Therefore, an analysis of proportion of patients testing annually in accordance with current WHO guidelines could not be performed.”*

Comment 13:

Investigators mention excluding known PLHIV who were already taking ART, but what about known PLHIV not yet on ART? Were they also excluded? If so, clarify.

Patients with self-reported HIV positive status who were not taking ART were included in the analysis. This would provide further opportunity for confirmatory testing and linkage to ART. This explanatory line has now been included in the methodology to make the manuscript easier for the reader to follow. Thank you for highlighting this.

*“Participants with known HIV-positive status who were already taking ART were excluded from the analysis. Those with self-reported HIV positive status who were not taking ART were included as this provides further opportunity for confirmatory testing and linkage to ART.”*

Results

Comment 14:

Are reasons for attendance to clinic, or clinic visit diagnosis available? Per above this may influence HIV testing as well.

Unfortunately, it would have been extremely challenging to collect and analyse participants’ presenting complaint and reason for clinic attendance, and so these data were not collected. As discussed above, because of poor quality routine collection of clinic attendances in the facility, it would not be possible to extract these data retrospectively or link to study data.

Comment 15:

Was clinic flow different for patients who reported TB symptoms? Would they have been tested for HIV at another clinic (i.e. during further TB workup?).

Bangwe Health Centre is able to offer comprehensive care including commencement of ART and anti-tuberculosis therapy. A line has been added to the methodology to inform the readers of this. With better understanding of the layout of Bangwe Health Centre we hope this is now an easier manuscript for the reader to follow.

*“PITC is recommended by the Malawi National HIV Programme for all attendances to an outpatient department, and was available at BHC, provided by Ministry of Health-accredited counsellors. ART and antituberculosis therapy were provided onsite at the HIV care clinic and TB clinic respectively.”*

Comment 16:

Were reasons test refusal among those offered testing recorded?

Due to limited time and number of research assistants questions were selected with binary options for answers. Qualitative answers were not obtained. This has now been explicitly stated in the methodology. Exploring these issues would of course have enhanced the manuscript. In the discussion we have included a new reference to qualitative research previously undertaken in this field which explores some of these reasons.

Comment 17:

Was time of last reported test recorded? Consider including in model.

Unfortunately, we did not collect these data, and has now been included as a limitation as discussed previously (Comment 12).

**Discussion**

Comment 18:

Mentions that majority of health centers use symptom-based PITC, as opposed to universal offer. Is this in agreement with Malawi PITC guidelines?

This is not in agreement with Malawian guidelines which state that all clinic attenders attending an outpatient appointment should be offered an HIV test. A statement confirming this has been added to the discussion. The discussion has been edited substantially to explain that these findings suggest non adherence to national guidelines and a need to develop new strategies for HIV testing. Thank you to the reviewer for highlighting this key issue.

*“This highlights lack of implementation of national guidelines which state that all patients attending an outpatient appointment should be offered an HV test. Previous qualitative research has revealed that health care workers felt more compelled to provide PITC in ANC than in outpatients departments. Healthcare workers also saw ART as a precious resource which should be reserved for those who were more unwell in outpatient departments.”*

Comment 19:

ROOT is mentioned for the first time in the Discussion. Suggest this be brought up earlier in the introduction to frame the reason to do the study.

In agreement with the reviewer, we have included a paragraph within the introduction which introduces strategies which may be potential solutions to low HIV testing offer rates. Following this introduction these two strategies for increasing testing (ROOT and semi-supervised oral fluid based self-testing) are then discussed further in the discussion.

*“New strategies for improving rates of HIV test offer within health facilities are essential to help reach the untested population. One strategy is routine opt-out (ROOT) HIV testing in which patients are offered an HIV test at each clinic visit unless they specifically refuse. A further is utilising semi-supervised oral fluid-based HIV testing kits within facilities.”*

Comment 20:

Discussion mentions WHO policy is in that setting with generalized epidemics that routine PITC should be offered to all with presumptive or diagnosed TB. Do the Malawian guidelines agree with this?

Malawian guidelines are in keeping with these WHO guidelines. A paragraph has been added to the discussion which outlines the Malawian guidelines. Also, in the discussion, it has been made clearer that our findings suggest suboptimal implementation of National guidelines. (Comment 6).

Comment 21:

The statistic that >50% of clinic attenders in the study reported TB symptoms is an important one and I’d suggest that this be mentioned in abstract.

The authors agree this is a key finding of the study. Highlighting the significant numbers of patients that may undergo missed opportunities for HIV testing and diagnosis. A line has been added to the abstract.

*“Presentation with any TB symptom was common for men (54.6%) and women (57.4%).”*

Comment 22:

Authors mention that only 44% of participants underwent exit interview as a limitation. One way to overcome this limitation is to include data from registers from the study period regarding TB symptoms (if available), HIV testing offer, and HIV test result. The latter 2 should be in the register.

Thank you for these comments. The clinic register in this setting cannot be proven to be accurate and therefore may result in inaccuracies in data. The study design used fingerprint bio identification to ensure that patient’s records were linked specifically for this reason.

Comment 23:

Given the data, what do the authors suggest are the next steps?

Thanks to the reviewers comments the discussion has been edited substantially to focus on strategies to improve HIV testing moving forward. This includes the implementation of ROOT into National guidelines as well as utilizing semi-supervised oral fluid self-testing within facilities.

**References**

Comment 24:

A minor comment, but please use consistent formatting/spacing in the references.

Thank you – we have corrected these.

Comment 25:

**Table 1**

Was any statistical testing done to see if there were differences between adult clinic attendees who had an exit interview or not. Suggest this is done. It appears that overall a lower proportion of participants who had a positive symptom screen underwent an exit interview.

Given the Bayesian analysis framework that downplays the importance of p-values based on non-significance hypothesis testing, we would prefer to not report p-values here, and instead emphasise differences in proportions as done in the manuscript.

Comment 26:

**Figure 1**

For the top row of graphs please label Men and Women if that is what the Y axis is.

Thank you. Figure 1 has now been updated. The figure has been displayed in a new format and an explanatory legend has been included.

*“Proportions of patients offered HIV testing. Stratified by those with TB symptoms (top row) and those without TB symptoms (bottom row).”*

**RESPONSE TO REVIEWER TWO’S COMMENTS**

Comment 1:

Authors have presented a nice study. They were interested to find out if adult patients seeking urgent care with TB-symptoms would receive PITC (as is recommended by WHO and Malawi National programmes).

Elegantly, they made their observations independent of the care offered at the health care facility (staffed by clinical officer, and nurses). Investigators positioned themselves at a registration desk for adult outpatients. Following verbal consent, identified patients through fingerprint biometrics and asked questions about age, HIV testing history, and TB symptoms.

Then, during exit interviews, 44% of adult patients were linked and patients were asked if an HIV test was offered, and if a test was taken.

Their data showed that contrary to their expectation, patients with TB symptoms were less likely to be tested than patients without TB patients. They also did statistical modeling to predict who would be more likely to be HIV tested.

The findings are clearly presented. This will be a useful contribution to the literature.

We thank the reviewer for their summary of the paper and positive feedback.

Comment 2:

I have the following questions that should be addressed in the revision.

Participants were asked if they had ever previously tested for HIV. Can authors clarify if they collected the date of the last HIV test? If they did not, then mention this as a study limitation. If they did, they can provide the proportion who tested in the last year (annual testing for adults of the general population is a WHO recommendation).

Thank you to the reviewer for these comments. Unfortunately, the time period since completion of last HIV test was not included in the participant exit interview. This has been added to the limitations section as suggested by the reviewer.

*“Thirdly patients were not asked the time since last HIV test in exit interview. Therefore an analysis of proportion of patients testing annually in accordance with current WHO guidelines could not be performed.”*

Comment 3:

Participants were asked their self-reported HIV status and ART status. HIV-positive patients on ART were excluded from the modeling. Can authors present the number of newly diagnosed patients (by self-report)? There should be patients who will have mentioned this, and the majority will/should have started same day treatment. Unless authors plan to report on self-reported test outcome - but they should say something about self-reported HIV testing at care seeking (as they asked about it). Is it possible that patients with a new HIV diagnosis who were started on ART were not interested to present the exit desk for interview? This should be mentioned as a limitation of this study.

The results of the study suggest that 5 patients were commenced on ART out of our sample of 455 patients who completed HIV testing. This finding is in keeping with the larger PROSPECT study in which this study was nested. This suggests a very high coverage of ART among clinic attenders in this health care facility, testament to the achievements of the Malawi National HIV Progrmme.

Comment 4:

Qualitative data (e.g. focus group discussions with providers) were not collected. I find this another important limitation of the study. It would have been so easy to present result back to providers and ask them why they through that younger patients, men, and patients without TB symptoms were more like to be HIV tested.

Thank you to the reviewer for these comments. Unfortunately, a qualitative study was not undertaken within this paper. This could have provided more understanding into the reasons for poor implementation of guidelines and should be included for further research.

In view of these comments we have included reference to qualitative research previously conducted in this field.

*“Previous qualitative research has revealed that health care workers felt more compelled to provide PITC in ANC than in outpatients departments. Healthcare workers also saw ART as a precious resource which should be reserved for those who were more unwell in outpatient departments.”*

Comment 5:

Other points that can be clarified / improved upon:

Discussion hinges on two arguments - leading to two proposed strategies. Poor performance of PITC should be improved through targeted testing and ROOT (routine opt out testing).

Poor performance: Vast numbers of patients with TB-symptoms are not tested. These are major missed opportunities. Rather than calling this "suboptimal" - be more bold in your conclusion! This is very poor implementation of existing guidelines. Why is that so? that remains unclear. Authors suggest that targeted PITC could be achieved through oral self-testing.

Thank you to the reviewer for this key comment. On refection we would agree that a firmer conclusion needs to be taken from this paper. We have therefore changed the terminology used both in the discussion and the conclusion to report that there is a lack of implementation of national guidelines.

We have also focused the discussion of the manuscript on rationale for why this occurred with reference to previous qualitative research in this field (Comment 4), and to focus on strategies for future improvement including expansion of discussion with regards to ROOT and oral fluid based self-testing.

Comment 6:

ROOT is presented as a possible if not best strategy. Is this likely? Given that current system under performs? Perhaps authors can more clearly distinguish the two strategies. Something like -- targeted PITC should be immediately implemented. ROOT is the preferred strategy but would require substantial resources.

The authors feel that implementation of ROOT into the National Guidelines can lead to a change of perception of need. If emphasis is placed on this strategy, as is in ANC guidelines, health care workers may be more inclined to offer testing in outpatient departments. References to previous qualitative research in this field (Comment 4 above) have explored reasons for lack of implementation of National Guidelines.

However, as the reviewer states our study shows lack of implementation of national guidelines and new testing strategies are needed to help reach the untested population. Therefore, we have placed greater emphasis on a second possible strategy of using semi-supervised oral self-testing within health facilities. A reference has been added highlighting previous successes of this strategy.

Small points:

Comment 7:

PITC HIV testing - misnomer - change to targeted HIV testing.

Thank you. Current WHO terminology recommends “Provider initiation HIV testing and counselling (PITC)”. https://www.who.int/hiv/topics/vct/PITC/en/. We have kept this as is.

Comment 8:

Is there a reference for ROOT?

A reference to ROOT and its success in Sub Saharan Africa has been included.

*“Silvestri DM, Modjarrad K, Blevins ML et al. A comparison of HIV detection rates using routine opt-out provider- initiated HIV testing and counseling versus a standard of care approach in a rural African setting. J Acquir Immune Defic Syndr. 2011 January 1; 56(1): e9–32”*

Comment 9:

Participants with a self-reported HIV testing history may drive them to request further HIV testing. Was this not explored in the exit interview? Were you offered an HIV test? Did you take an HIV test? or Did you ask for an HIV test yourself?

This was a new finding within the study that was unexpected. The question “did you ask for an HIV test yourself?” was not included in the study protocol. However, this question can be included in further research.

Comment 10:

First paragraph discussion - last sentence. Optimism can be taken from an increase of 89.9% to 90.8%. There is either a typo in the stats, but i find this a minimal increase not worth discussing and no reason for optimism.

This sentence has now been removed from the manuscript.

Comment 11:

Last sentence of introduction (stratification by sex) reads as a methods sentence and is better placed in methods.

This sentence has been moved to the methods section.

Comment 12:

Approximately 1 in 4 patients is offered testing. And of those offered, 4 out of 5 accept HIV testing. Is it clear why a substantial number of patients refuse testing?

Rationale for participant’s non-completion of HIV testing was not evaluated in this study. A proportion of these patients may not have refused testing but instead were unable to undergo testing due to limited numbers of health-care workers or test kits.

*“These stated limitations may account for the population of patients in our study who were offered HIV testing but did not undergo a test.”*
