# Supplementary material for: Provider-initiated HIV testing and TB screening in the era of universal coverage: Are the right people being reached? A cohort study in Blantyre, Malawi
Source: PLoS One. 2020 Aug 13;15(8):e0236407. doi: 10.1371/journal.pone.0236407 (PMC7425889; doi:10.1371/journal.pone.0236407)
Supplement: Attachment — Submitted filename: Response to Reviewers June 2020.docx [file pone.0236407.s003.docx]

Dr Luke Mair MBChC, DTM&H
Department of Clinical Sciences,
Liverpool School of Tropical Medicine,
Pembroke Place,
Liverpool,
L3 5QA

Email: [Luke.mair1@nhs.net](mailto:Luke.mair1@nhs.net)
Mob: +447969239797

Dear Editors and Reviewers,

**RE: Provider-initiated HIV testing and TB screening in the era of universal coverage: are the right people being reached? A cohort study in Blantyre, Malawi**

I write on behalf of my co-authors to thank the reviewers for their careful second review, and appreciate the opportunity to respond with a revised submission. Please see a point-by-point response below.

We hope that with the changes made as suggested by the reviewers this manuscript can be published and provide motivation for a change in HIV testing policy.

Yours sincerely,

Dr Luke Mair

**RESPONSE TO REVIEWER 1’s COMMENTS**

**Comment 1**:

Abstract

Clarify that this was among clinic attendees with acute illness. Dates are typically spelled out, but leave this style issue to the editors. Could consider simplifying abstract with regards to the categories of symptoms- since participants with and without symptoms the proportion offered testing was the same, why not just say among participants with any TB symptoms (especially since there appeared to be no difference among each specific symptom).

Thank you to the reviewer for these comments. In response additional clarification has been added to the abstract that these were participants with acute illness.

*“In a prospective cohort study in Bangwe primary care clinic, Blantyre, Malawi, all adults (18 years or older)* ***presenting with an acute illness*** *were screened for TB symptoms (cough, fever, night sweats, weight loss).”*

As suggested by the reviewer we have removed the percentages for each individual condition to make the abstract more readable. We have included an additional line in the text to explain that there was no difference between each symptoms.

*“The proportions offered testing were similar among participants with and without TB symptoms (any TB symptom: 29.0% vs. 25.7%). This was consistent for each individual symptom; cough, weight loss, fever and night sweats.”*

**Comment 2**:

Agree with the conclusion of the Abstract.

Thank you for these positive comments.

**Comment 3:**

Introduction

Perhaps mention that Malawi is not one of the 7 countries to meet this 90/90/90 target.

The authors agree that providing additional clarity here will add clarity for the reader. Therefore an additional sentence is added.

*“Malawi is among the large proportion of countries still striving to reach these targets.”*

**Comment 4:**

Add reference for ROOT and oral testing

Thank you. We have included references for each as follows.

**ROOT**: Silvestri et al. A comparison of HIV detection rates using routine opt-out provider-initiated HIV testing and counseling versus a standard of care approach in a rural African setting. J Acquir Immune Defic Syndr. 2011

**Oral testing**: Choko et al. 2015. Uptake, Accuracy, Safety, and Linkage into Care over Two Years of Promoting Annual Self-Testing for HIV in Blantyre, Malawi: A Community-Based Prospective Study. PLoS Med.

**Comment 5:**

Methods

-Provide reference of the Malawi national HIV Programme guidelines.

Thank you. This reference has now been included.

*“Reference 3: Ministry of Health of Malawi. Malawi HIV Testing Services Guidelines. Lilongwe; 2016”*

**Comment 6:**

-Be explicit that patients with known HIV on ART were excluded in the Methods.

Thank you. We have clarified this in the Methods:

*“Participants with known HIV-positive status who were already taking ART were excluded from the analysis.”*

**Comment 7:**

-Research assistant should be lower case I believe.

We are in agreement that these should be described in lower case and this change has been made.

**Comment 8:**

-What does opportunistic manner mean?

We have provided further clarity to this methodology as below.

*“Research assistants were also based at the two clinic outpatient department exits, where they identified adults leaving the department and invited them to participate in an exit interview. The layout of the clinic is such that all participants exiting the clinic would pass the research assistants. Though researchers tried to selected as many patients as possible, due to limitations in the number of research assistants not all participants completed exit interview.”*

**Comment 9:**

-Please be consistent with references either before or after a period.

This has been addressed and references now always appear after periods. Thank you.

**Comment 10:**

-Not sure why there is a page break after Data and reproducability and Results?

This has been removed.

**Comment 11:**

Results

-Provide % for the 276 attenders who were HIV+ on ART that were excluded.

This has now been included in the manuscript.

*“A total of 276 (11.5%) clinic attenders reported that they were HIV positive and taking ART and were exclude from regression modelling.”*

**Comment 12:**

-Strikingly low offer of HIV testing (27.6%). (comment only)

-Striking that participants with a previous history of TB weren’t offered HIV testing. (comment only).

Thank you for these comments. These show the suboptimal rates of testing offer in high risk populations which the manuscript aims to highlight.

**Comment 13:**

-Among those offered but not tested, what was the reason?

Rationale for participant’s non-completion of HIV testing was not evaluated in this study. A proportion of these patients may not have refused testing but instead were unable to undergo testing due to limited numbers of health-care workers or test kits.

*“These stated limitations may account for the population of patients in our study who were offered HIV testing but did not undergo a test.”*

**Comment 14:**

Discussion

-Please be careful with punctuation (2 periods at end of firs sentence in Discussion)

This has been addressed.

**Comment 15:**

-Seems important to point out in the first paragraph of the discussion that this low rate of testing is despite the national guideline recommendations.

Thank you for this suggestion. A sentence has been added to the first paragraph of the discussion to highlight this key issue

*“This highlights lack of implementation of national guidelines, which state that all patients attending an outpatient appointment should be offered an HV test.”*

**Comment 16**:

-ROOT already defined in Introduction, consider using only abbreviation here.

Corrected as recommended

**Comment 17:**

-Were there test kit stockouts during the time? It’s mentioned as a potential reason, but no further discussion.

Thank you for this suggestion. No, there were no stock-outs during the study period.

**Comment 18:**

-Although oral testing is a fantastic idea- why do the authors think this would increase testing? What does semi-supervised mean in regards to oral testing?

A large body of evidence is now supporting HIV self-testing as a complementary strategy to increase uptake of HIV testing. Semi-supervised means that a health worker is in attendance, or available, should the patient require additional help in performing or interpreting the test themselves.

**Comment 19:**

References

-Still has inconsistent formatting with references (some with hanging indent, some not).

Thank you. This has been addressed.

**Comment 20:**

-I’m a bit surprised that one of the national guideline referenced is from 2012.

Thank you. These are the most recent TB screening guidelines. WHO is currently updating its TB screening guidelines, and we anticipate updated Malawi guidelines thereafter.

**Comment 21:**

Table 1

-Please use consistent capitalization for row headers.

This has been addressed

**Comment 22:**

-What are the numbers separated by a comma? Are they IQR? Ranges? Please specific in the table.

Thank you. These are ranges (min and max), as indicated in the table row legend.

**Comment 23:**

Table 2

Define WAIC

Thank you. WAIC is the Watanabe-Akaike information criteria. This is a standard information criteria for assessing model fit.

**RESPONSE TO REVIEWER TWO’S COMMENTS:**

**Comment 1**

Authors may want to check the following added sentence in their discussion:

"Qualitative research has suggested that patients often wish to know their status due a period of health and therefore may be motivated to test themselves"

Not clear what is meant by a period of health

This sentence has been changed and now reads.

*“Qualitative research has suggested that patients who noticed a decline in their health, may wish to know their status and therefore may be motivated to test themselves.”*
